# Pancreatic stellate cell-induced gemcitabine resistance in pancreatic cancer is associated with LDHA- and MCT4-mediated enhanced glycolysis

**DOI:** 10.1186/s12935-023-02852-7

**Published:** 2023-01-19

**Authors:** Manoj Amrutkar, Kjersti Berg, Aina Balto, Miguel G. Skilbrei, Anette V. Finstadsveen, Monica Aasrum, Ivar P. Gladhaug, Caroline S. Verbeke

**Affiliations:** 1 Department of Pathology, Oslo University Hospital Rikshospitalet, Oslo, Norway; 2 Department of Pharmacology, Institute of Clinical Medicine, University of Oslo, Oslo, Norway; 3 Department of Hepato-Pancreato-Biliary Surgery, Institute of Clinical Medicine, University of Oslo, Oslo, Norway; 4 Department of Hepato-Pancreato-Biliary Surgery, Oslo University Hospital Rikshospitalet, Oslo, Norway; 5 Department of Pathology, Institute of Clinical Medicine, University of Oslo, Oslo, Norway

**Keywords:** Pancreatic cancer, Pancreatic stellate cells, Glycolysis, Gemcitabine sensitivity

## Abstract

**Background:**

Profound resistance to chemotherapy remains a major challenge in achieving better clinical outcomes for patients with pancreatic ductal adenocarcinoma (PDAC). Recent studies indicate that gemcitabine (GEM) resistance is promoted both by pancreatic stellate cells (PSCs) and through increased glycolysis. However, it remains unknown whether PSCs affect GEM sensitivity via glycolytic regulation.

**Methods:**

Human pancreatic cancer cell (PCC) lines (BxPC-3, Capan-2, HPAF-II, Mia PaCa-2, Panc-1, SW-1990) were exposed to three different PSC-conditioned media (PSC-CM; PSC-1, PSC-2, HPaSteC), following either pre-treatment with glycolysis inhibitor NV-5440 or transfection for transient silencing of key glycolytic regulators (LDHA and MCT4). Proliferation, glucose transport, extracellular lactate, and GEM sensitivity were assessed. Protein expression was determined by Western blot and immunostaining. Moreover, secreted proteins in PSC-CMs were profiled by mass spectrometry (MS).

**Results:**

While exposure to PSC-CMs did not affect glucose transport in PCCs, it increased their lactate release and proliferation, and reduced the sensitivity for GEM. Both NV-5440 treatment and transient silencing of LDHA and MCT4 inhibited these PSC-induced changes in PCCs. MS analysis identified 688 unique proteins with differential expression, of which only 87 were common to the three PSC-CMs. Most PSC-secreted proteins were extracellular matrix-related, including SPARC, fibronectin, and collagens. Moreover, exposure to PSC-CMs increased the phosphorylation of ERK in PCCs, but the treatment of PCCs with the MEK/ERK inhibitor PD98059 resulted in a reduction of PSC-CM-induced glycolysis and improved GEM sensitivity.

**Conclusions:**

The study findings suggest that PSC-secreted factors promote both glycolysis and GEM resistance in PCCs, and that glycolysis inhibition by NV-5440 and blocking of ERK phosphorylation by PD98059 protect PCCs from PSC-CM-induced loss of GEM sensitivity. Taken together, PSCs appear to promote GEM resistance in PDAC via glycolysis. Thus, targeting glycolysis may improve the effect of chemotherapy in PDAC.

**Supplementary Information:**

The online version contains supplementary material available at 10.1186/s12935-023-02852-7.

## Background

Pancreatic ductal adenocarcinoma (PDAC) is one of the most lethal solid cancers, the 5-year survival rate remaining below 10% [1, 2]. Surgery is the only potential cure, however, the majority of patients with PDAC (> 80%) are inoperable at diagnosis due to advanced disease. For these patients, chemotherapy is the standard of care, and regimens include gemcitabine (GEM), either as monotherapy or combined with nab-paclitaxel, or FOLFIRINOX. Despite suboptimal clinical benefits, GEM remains a cornerstone of treatment for PDAC [3, 4]. Treatment failure due to profound drug resistance is a significant cause of the poor prognosis of PDAC [5].

The characteristic stroma-rich tumor microenvironment (TME) and its interactions with the cancer cells are major contributors to chemoresistance in PDAC [6, 7]. Particularly, pancreatic stellate cells (PSCs, also called cancer-associated fibroblasts), which represent the largest cellular component of the TME, were shown to promote GEM resistance in pancreatic cancer cells (PCCs) [8–12]. Moreover, PSCs are increasingly recognized for their support in PDAC progression via reprogramming of the cancer cell metabolism [13–15]. Enhanced glycolysis is one of the main metabolic changes observed in PDAC, which fulfills the high energy demands of fast-proliferating cells by supplying biosynthetic building blocks [16]. Moreover, PSCs were recently shown to contribute to glycometabolic alterations in PDAC through a ‘reverse Warburg effect’. In the latter, cancer cells induce oxidative stress-mediated aerobic glycolysis in neighboring PSCs, which leads to increased production of high-energy fuels such as lactate, pyruvate, ketone bodies, and fatty acids that are useful nutrient sources for the cancer cells [17, 18].

It is only recently that the potential association between glycometabolic alterations, GEM sensitivity in PDAC, and tumor growth have been investigated. Increased glycolytic flux was shown to induce glucose addiction in cancer cells, which promotes pyrimidine biosynthesis. This subsequently increases intracellular deoxycytidine levels and contributes to reduced GEM efficiency via competitive inhibition [19]. Moreover, enhanced glycolytic activity was shown to promote tumor growth and reduce GEM sensitivity in PDAC [20–22]. Reduced tumor growth and metastasis were observed following inhibition or blocking of the key glycolytic regulators HK, PFK1, and LDHA [23–25].

Taken together, growing evidence indicates that both tumor growth and GEM sensitivity are affected by altered glycometabolism in PDAC. However, there is a lack of evidence as to whether glycolysis and GEM sensitivity in PCCs are linked and whether both processes are mediated by interactions between the PSCs and cancer cells. To this end, this study investigates the impact of PSC-conditioned medium (PSC-CM) on glucose transport, glycolysis, and GEM sensitivity in PCCs.

## Methods

### Cell lines, culture and maintenance

Six PCC lines—BxPC-3, Capan-2, HPAF-II, Mia PaCa-2, Panc-1, SW-1990—used in this study, were purchased from ATCC (Manassas, VA, USA), see Additional file 1: Table S1 for details. Two primary human PSC cultures (PSC-1, PSC-2) were established with the outgrowth method from surgically resected treatment-naïve and neoadjuvantly treated PDAC, respectively [26, 27]. Clinicopathological features of the source cancers for both PSC cultures are provided in Additional file 1: Table S2. Human pancreatic fibroblasts HPaSteC derived from the human pancreas of a 22-week-old, fetal, non-diseased, male donor, was purchased from ScienCell Research Laboratories (#3830; San Diego, CA, USA).

Both PCCs and PSCs were maintained at 37 °C with 5% CO_2_ in a normal growth medium, i.e., Dulbecco’s modified Eagle’s medium (DMEM) containing 4.5 g/L D-glucose (GlutaMAX™, #31966047), supplemented with 10% FBS (#10500064), and 1% each of penicillin–streptomycin (#15140122) and amphotericin B (#15290026). During the experiments, cells were maintained in serum-free DMEM (SFM), or in a low-glucose medium, i.e., DMEM containing 1.0 g/L D-glucose (GlutaMax™, #21885025). All culture media and supplements were purchased from Thermo Fisher Scientific (Waltham, MA, USA). Cell cultures were routinely checked for mycoplasma using MycoAlert™ Mycoplasma Detection Kit (#LT07-703; Lonza, Basel, Switzerland). Cell lines were authenticated using short tandem repeat (STR) profiling (Eurofins Genomics, Ebersberg, Germany).

### Preparation of PSC-conditioned medium (PSC-CM)

The PSC-CMs were obtained as described previously [8]. Prior to the preparation of PSC-CMs, all three PSCs were checked for the expression of α-smooth muscle actin (α-SMA) and vimentin using immunostaining (Additional file 2: Fig. S1). Briefly, individual PSC cultures grown to sub-confluence in 100-mm Falcon™ Standard Tissue Culture Dish (#08-772E; Fisher Scientific, Oslo, Norway) were thoroughly washed with PBS and subsequently incubated with SFM (~16 ml per dish) at 37 °C for 48 h. The culture medium was collected and centrifuged at 5000 *g* for 10 min, and the supernatant (PSC-CM; ~15 ml) was collected and stored at − 20 °C until further use.

### Experimental design

PCCs grown in PSC-CMs or SFM for 72 h were assessed for changes in cell morphology, proliferation, glucose transport, extracellular lactate release, and GEM sensitivity. In addition, PCCs pre-treated with an inhibitor of selective class I glucose transporter—NV-5440 (#SML2781; Sigma-Aldrich, St Louis, MO, USA), or an inhibitor of MEK/ERK—PD98059 (#S1177; Calbiochem, La Jolla, CA) or PCCs transfected with targeted siRNAs against LDHA and MCT4 were also investigated. Except for protein lysate collections, PCCs seeded in 96-well microplates (Corning® #CLS3596; Sigma-Aldrich) at a density of ~5000 cells per well were used in all experiments. Experimental procedures were followed as described below.

### Assessment of cell morphology, viability, proliferation, and chemosensitivity

For morphological assessment, PCCs were stained with crystal violet solution (#94448; Sigma-Aldrich) containing 20% methanol, for 20 min at room temperature. Thereafter, cells were washed with tap water until excessive crystal violet was removed, and images were captured under the light microscope (Zeiss, Oberkochen Germany). To determine cell viability, cells were incubated with MTT reagent (0.25 mg/ml; #M5655; Sigma-Aldrich) for 4 h, and the conversion of MTT to formazan crystals by metabolically viable cells was evaluated using a spectrophotometer at 570 nm, as described previously [28]. Cell proliferation was determined by measuring BrdU incorporation into actively proliferating cells using the BrdU Cell Proliferation ELISA kit (#ab126556; Abcam, Cambridge, UK), according to the manufacturer’s instructions. For chemosensitivity assessment, PCCs treated with GEM at a final concentration of 10 µM for 48 h, were investigated for drug-induced cytotoxicity using MTT assay, as described previously [8].

### Glucose transport and lactate release

Cells were washed with PBS and incubated for the indicated duration with Krebs Ringer HEPES buffer (KRH; 100 µl per well) containing 10% start solution. The start solution consisted of a mixture of 0.5 µCi [^3^H]-2-deoxy-D-glucose (2.6%; #NET238C; PerkinElmer, Waltham, MA, USA), 1 mM 2-deoxy-D-glucose (26%; #D8375; Sigma-Aldrich) and the remaining volume of PBS (71.4%). The reaction was terminated by 10 min incubation with the stop solution (5 µl per well), which consisted of a mixture of 0.4 mM Phloretin (4.2%; #P7912; Sigma-Aldrich), methanol (20%; Sigma-Aldrich) and PBS (75.8%). Lastly, cells were washed with PBS and subsequently lysed by incubation with 0.2 M NaOH (100 µl per well) for 10 min on a rotating shaker at room temperature. One half of each lysate was transferred to a scintillation vial (Sarstedt, Germany) containing 4 ml Ultima Gold solution (PerkinElmer). Cell-associated radioactivity, measured in counts per minute (CPM), was determined using a liquid scintillation counter. Glucose uptake was calculated by normalizing CPM to the protein amount in each sample. For the assessment of extracellular lactate levels, cell culture supernatants were analyzed using Glycolysis Cell-Based Assay Kit (#600450; Cayman Chemicals, Ann Arbor, MI, USA), according to the manufacturer’s instructions. The amount of lactate was adjusted to the protein content in each well. The other half of each cell lysate was used to determine the protein content by using the Bradford assay.

### Transient gene silencing

An siRNA-mediated gene silencing approach was used to achieve transient silencing of LDHA and MCT4 in PCCs. Cells were transfected with targeted siRNAs with sequences listed in Additional file 1: Table S3, using Lipofectamine RNAiMAX (#13778150; Invitrogen, Waltham, MA, USA), as described previously [28]. Cells transfected with non-targeting scrambled siRNA (#AM4611; Thermo Fisher Scientific) were used as a negative transfection control (NTC). Transfection efficiency was determined by protein expression analysis using western blot and immunostaining. Optimal transfection conditions were determined using nuclear incorporation of siGLO Green Transfection Indicator (#D-001630–01; Dharmacon, Lafayette, CO, USA).

### Protein expression analysis

Protein expression was analyzed using western blot and immunostaining, as described previously [28, 29]. For western blot, either PCCs seeded in 6-well plates (~100 000 cells per well) cultured to confluence in a normal growth medium, or sub-confluent PCCs incubated with SFM or PSC-CM for 72 h were used. Protein extracts were obtained by cell lysis using Laemmli buffer. Proteins separated by electrophoresis were transferred to nitrocellulose membranes and subsequently incubated with respective antibodies. For immunostaining, cells fixed in 4% paraformaldehyde were stained with respective antibodies, and images were captured using FLoid™ Cell Imaging Station (#4471136; Thermo Fisher Scientific). Antibody information is provided in Additional file 1: Table S4.

### Mass spectrometry-based proteomic analysis of PSC-CMs

The PSC-CM from PSC-1, PSC-2, and HPaSteC in triplicates of 5 ml for each culture, were analyzed for secreted proteins, using mass spectrometry (MS), as described previously [29]. Each PSC-CM aliquot was reduced to 5% of the original volume by using a 10 kDa cut-off Amicon Ultra centrifugal filter. Subsequently, proteins were reduced, alkylated, and digested overnight using trypsin (Promega, Madison, WI, USA). Peptides were desalted and concentrated before submission to MS. Each peptide mixture was analyzed by nEASY-LC coupled to QExactive Plus (ThermoElectron, Bremen, Germany) with EASY Spray PepMap®RSLC column (C18, 2 µm, 100 Å, 75 µm × 50 cm). Proteome Discoverer 2.1 (Thermo Fisher Scientific) and Mascot 2.6 (MatrixScience, London, UK) search engine were used for protein identification. The following search criteria were used for Mascot searches: trypsin digestion with two missed cleavage allowed, carbamidomethyl (C) as fixed modification and Acetyl (N-term), Gln- > pyro-Glu (N-term Q), Oxidation (M) as dynamic modifications. The parent mass tolerance was 10 ppm, and MS/MS tolerance was 0.1 Da. Database searches were performed using SwissProt for human entries, supplemented with known contaminants provided by MaxQuant. All reported protein identifications were statistically significant (*p* < 0.05) in Mascot and filtered in Proteome Discoverer for at least medium confidence identifications. The list of identified proteins was subjected to the Kyoto Encyclopedia of Genes and Genomes (KEGG) pathway analysis. Gene ontology (GO) analysis was conducted using the DAVID bioinformatics tool.

### Statistical analysis

All values are expressed as mean ± SEM. The statistical analysis of the results was performed using GraphPad Prism 6 software and Microsoft Excel 2016, by an unpaired two-tailed Student’s t-test with a value of *p* < 0.05 being considered statistically significant.

## Results

### Time-dependent glucose transport in PCCs

**Fig. 1:**
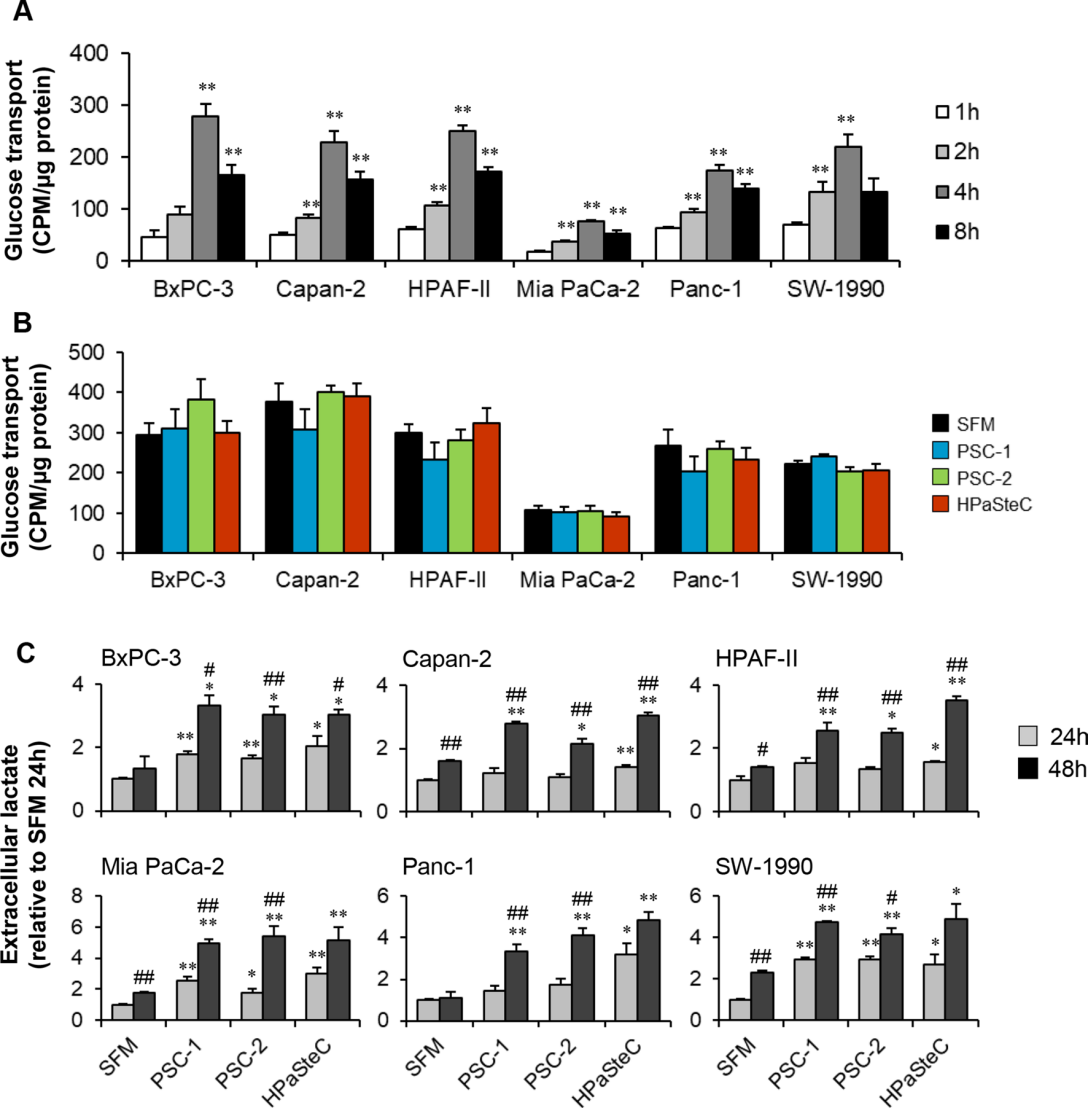
Assessment of PCC glucose transport and lactate release. **A** Time-dependent glucose transport in PCCs at baseline. **B-C** PCCs pre-incubated for 72 h with SFM or three different PSC-CMs (PSC-1, PSC-2, and HPaSteC) were investigated for **B** glucose transport at 4 h and **C** extracellular lactate at 24 h and 48 h. For **C**, cells were maintained in low-glucose SFM during lactate release assessment. Data presented as mean ± SEM of 3–4 replicates. For **A** *p < 0.05, **p < 0.01 comparing 2 h, 4 h, and 8 h with 1 h. For **C** *p < 0.05, **p < 0.01 comparing SFM with PSC-CM at 24 h or 48 h, and ^#^*p* < 0.05, ^##^*p* < 0.01 comparing 24 h with 48 h in both SFM and PSC-CM. *PCC* pancreatic cancer cell, *PSC-CM* pancreatic stellate cell-conditioned medium, *SFM* serum-free DMEM

To investigate whether glucose transport in PCCs is time-dependent, cells exposed to [^3^H]-glucose at baseline were assessed for their intracellular levels at four different time points: 1, 2, 4, and 8 h (Fig. 1A). Across six PCC lines, a time-dependent increase in glucose transport was observed, which reached its maximum at 4 h. Compared to 1 h, glucose transport was higher at all other time points investigated, while intracellular glucose levels at 8 h were lower than at 4 h (Fig. 1A). Moreover, glucose transport was heterogeneous among the PCC lines, with BxPC-3 and Mia PaCa-2 showing the highest and lowest intracellular glucose levels at 4 h, respectively. Moreover, Mia PaCa-2 showed overall lower glucose transport compared to the other PCCs (Fig. 1A).

### Unaltered glucose transport and higher lactate release by PCCs upon exposure to PSC-CMs

PCCs incubated with three different PSC-CMs (PSC-1, PSC-2, and HPaSteC) for 72 h were investigated for changes in morphology, glucose transport, and extracellular lactate release. Assessment of crystal violet-stained PCCs revealed no notable change in morphology following exposure to PSC-CMs as compared to SFM (Additional file 2: Fig. S2). No significant change in glucose transport, assessed at 4 h, was observed in PCCs incubated with PSC-CMs compared to SFM controls (Fig. 1B). For assessment of extracellular lactate release, PCCs pre-incubated for 72 h with PSC-CM or SFM were maintained in low-glucose DMEM for 48 h. Lactate content in the medium was measured at 24 h and 48 h. All six PCC lines showed a significantly higher lactate release at 48 h by cells incubated with any of the three PSC-CMs as compared to SFM (Fig. 1C). Moreover, lactate release at 48 h was significantly higher than at 24 h in all PCCs and for all three PSC-CMs. When comparing the impact of the PSC-CMs on lactate release by the PCCs at 48 h, the following ranking was observed: HPaSteC > PSC-1 > PSC-2 for Capan-2, HPAF-II, and SW-1990; and HPaSteC > PSC-2 > PSC-1 for Panc-1. In BxPC-3 and Mia PaCa-2, the three PSC-CMs had a similar effect (Fig. 1C). Similarly, the impact of PSC-CM on lactate release by the PCCs at 24 h was also heterogeneous. Compared to SFM, lactate release at 24 h was significantly higher in BxPC-3, Mia PaCa-2, and SW-1990 following exposure to any of the three PSC-CMs, whereas in Capan-2, HPAF-II, and Panc-1 only exposure to HPaSteC had a similar effect (Fig. 1C).

### Increased proliferation and reduced GEM sensitivity in PCCs exposed to PSC-CMs

**Fig. 2:**
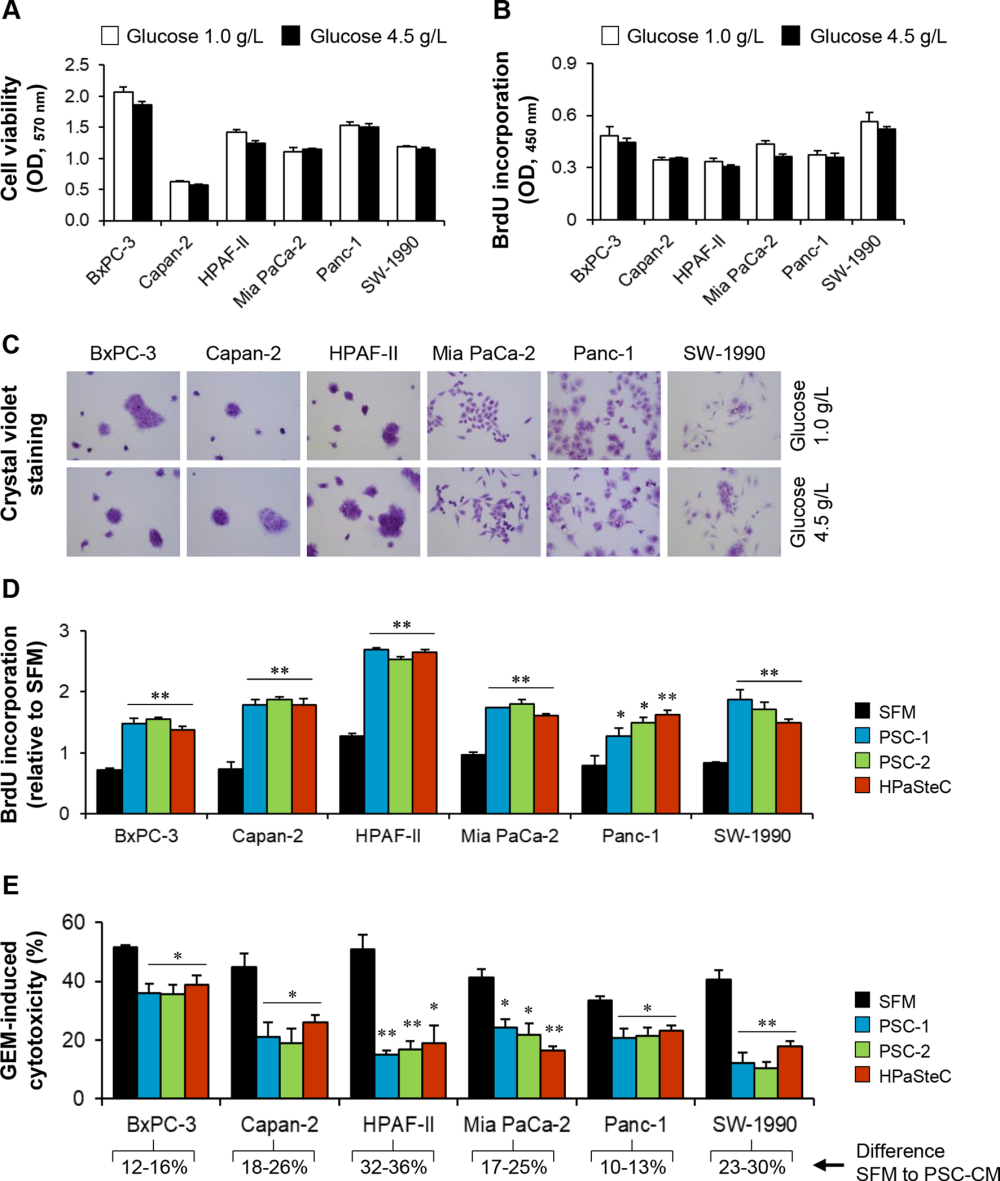
Assessment of growth and GEM sensitivity in PCCs. PCCs grown in media containing 1.0 g/L or 4.5 g/L glucose were assessed for **A** viability using MTT assay, **B** proliferation using BrdU incorporation assay, and **C**. morphology by crystal violet staining. Next, PCCs incubated for 72 h with SFM or three different PSC-CMs (PSC-1, PSC-2, and HPaSteC) were investigated for changes in **D** proliferation and **E** sensitivity to GEM (10 µM) following 48 h treatment. Data presented as mean ± SEM of four replicates. For **D**, **E**, *p < 0.05, **p < 0.01 comparing PSC-CM with SFM. GEM, gemcitabine; PCC, pancreatic cancer cell; PSC-CM, pancreatic stellate cell-conditioned medium; SFM, serum-free DMEM

To investigate the impact of reduced glucose availability on PCC growth, cells grown for 72 h in a medium containing low glucose (1.0 g/L) or normal glucose (4.5 g/L), supplemented with 1% FBS, were evaluated for cell viability, proliferation, and morphology. Cell viability (Fig. 2A) and proliferation (Fig. 2B), assessed by MTT assay and BrdU incorporation, respectively, both remained unaltered in the six PCC lines, irrespective of the level of glucose exposure. Similarly, cell morphology, evaluated by crystal violet staining, was not notably different between PCCs grown in a medium containing low or normal glucose (Fig. 2C). Next, the impact of PSCs on PCC proliferation was determined following the exposure of PCCs to PSC-CMs for 72 h. All six PCC lines showed a significant increase in proliferation (1.6- to 2.6-fold, *p* < 0.05) upon exposure to any of the three PSC-CMs as compared to SFM (Fig. 2D). Notably, the magnitude of increase in proliferation was similar across PCCs when the three PSC-CMs were compared (Fig. 2D). To determine the impact of PSCs on GEM sensitivity, drug-induced cytotoxicity was measured following 72 h incubation with PSC-CMs or SFM, and subsequent treatment with 10 µM GEM for 48 h. Compared to SFM, PCCs incubated with any of the three PSC-CMs showed a significant reduction in GEM-induced cytotoxicity (Fig. 2E). Comparison of the effect between the various PSC-CMs revealed no clear pattern, although a differential response to cytotoxic actions of GEM was observed in the PCC lines. In SFM, GEM sensitivity was highest and lowest in BxPC-3 and Panc-1, respectively. In contrast, PSC-CM-induced reduction in GEM sensitivity was most prominent in HPAF-II (> 30%), followed by SW-1990, Capan-2, and Mia PaCa-2 (17–30%), and it was smallest in Panc-1 and BxPC-3 (10–16%; Fig. 2E).

### Higher expression of key glycolytic regulators in PCCs exposed to PSC-CM

**Fig. 3:**
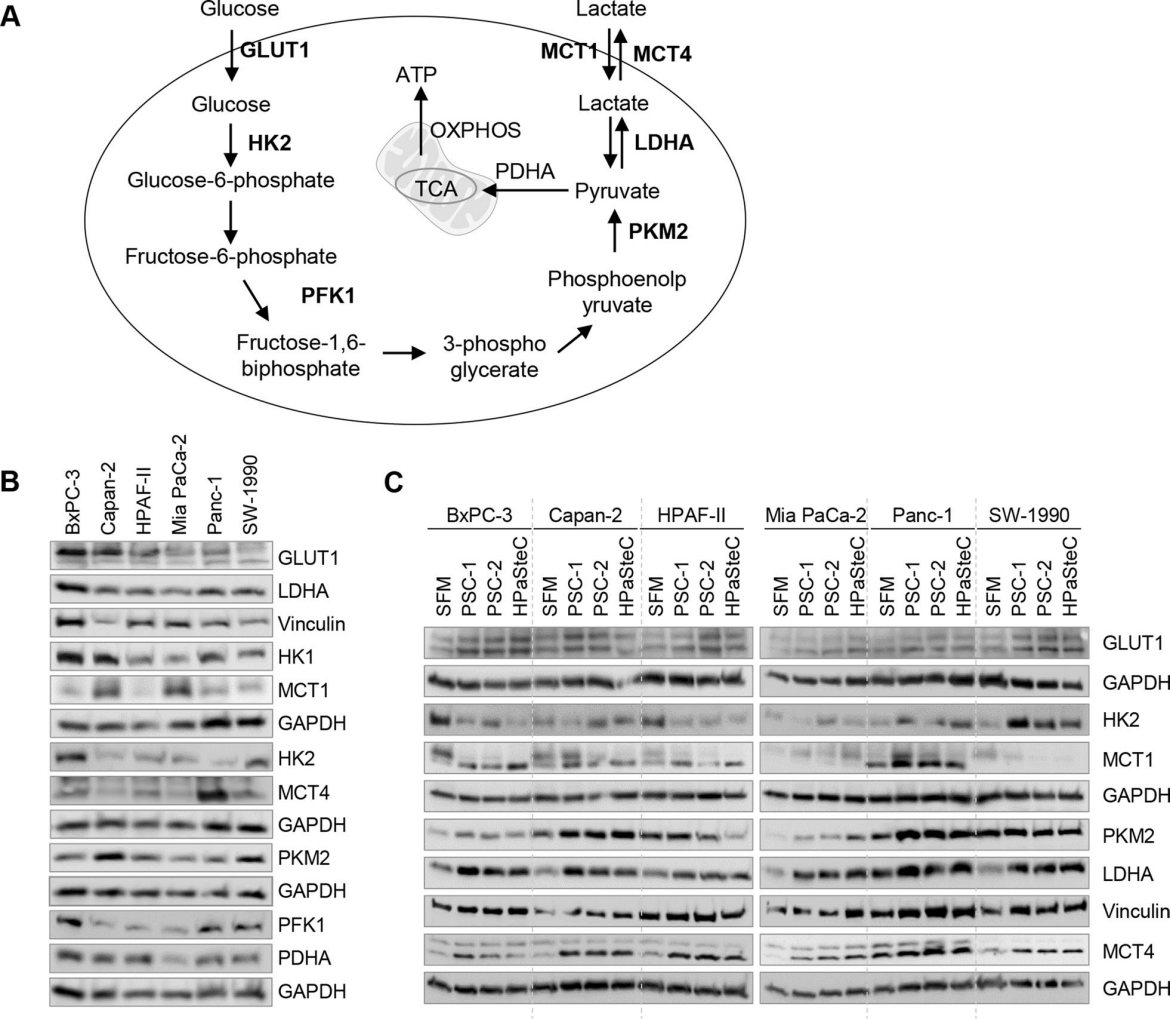
Expression analysis of glucose metabolism pathway markers. **A** Schematic presentation of glucose to lactate conversion in PCCs. Cell lysates collected from **B** PCCs at baseline, and **C** PCCs incubated for 72 h with SFM or three different PSC-CMs (PSC-1, PSC-2, and HPaSteC) were analyzed by Western blot for expression of the indicated proteins. GAPDH and vinculin were used as loading controls. GAPDH, glyceraldehyde 3-phosphate dehydrogenase; GLUT1, glucose transporter 1; HK (1, 2), hexokinase 1, 2; MCT (1, 4), monocarboxylate transporters 1, 4; LDHA, lactate dehydrogenase A; PCC, pancreatic cancer cell; PDH, pyruvate dehydrogenase; PFK1, phosphofructokinase 1; PKM2, pyruvate kinase M2; PSC-CM, pancreatic stellate cell-conditioned medium; SFM, serum-free DMEM

A schematic representation of the glucose metabolism pathway is provided in Fig. 3A. Considerable heterogeneity in the expression of key glucose metabolism pathway markers was observed among the six PCCs at baseline (Fig. 3B). The following pattern was observed: GLUT1, LDHA, PFK1, and PDHA were lowest in Mia PaCa-2 and highest in BxPC-3; PKM2 was highest in Capan-2; hexokinases HK1 and HK2 both were highest in BxPC-3; MCT1 was highest in Mia PaCa-2 and lowest in HPAF-II; MCT4 was highest in Panc-1 (Fig. 3B). Next, PCCs exposed to PSC-CMs for 72 h were investigated for expression of GLUT1, HK2, PKM2, LDHA, MCT1, and MCT4 (Fig. 3C). Compared to SFM, exposure to PSC-CMs resulted in a significantly higher expression of LDHA and MCT4 in all six PCC lines (Fig. 3C). A higher expression level of GLUT1 and PKM2 was observed in some of the PCCs (GLUT1: BxPC-3, Capan-2, HPAF-II, SW-1990; PKM2: BxPC-3, Capan-2, Mia PaCa-2, Panc-1). Expression of HK2 and MCT1 was highly variable both among the PCCs at baseline (SFM) and following exposure to the three different PSC-CMs (Fig. 3C).

### PSC-CM-induced loss of GEM sensitivity in PCCs restored by the potent glycolysis inhibitor NV-5440

**Fig. 4:**
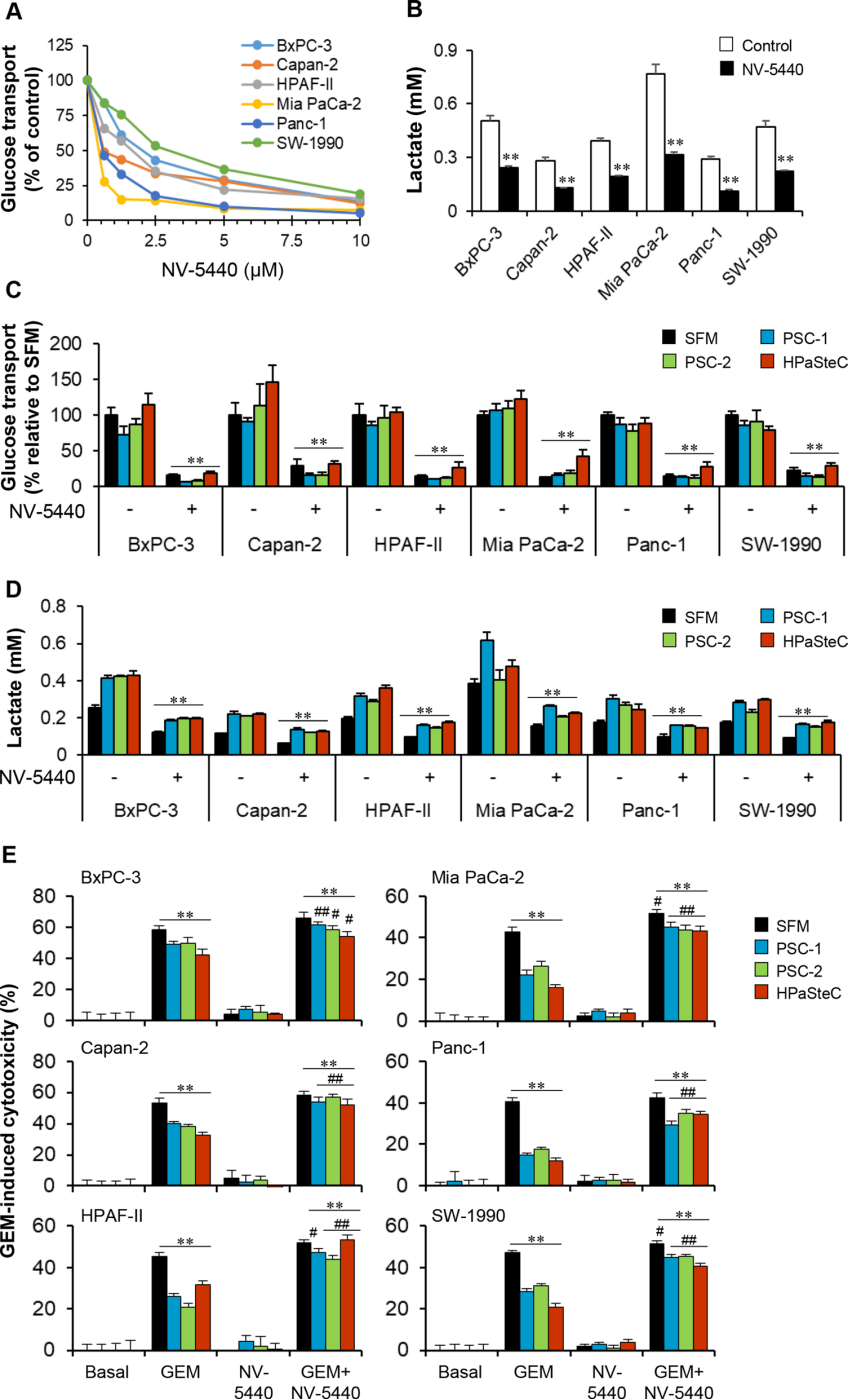
Effect of NV-5440 on glucose transport, glycolysis, and GEM sensitivity in PCCs. **A** Dose-dependent inhibition of glucose transport in PCCs by NV-5440. **B** Impact of NV-5440 (10 µM) on extracellular lactate release by PCCs. Assessment of **C** glucose transport at 4 h and **D** lactate release at 48 h following 2 h treatment with NV-5440 in PCCs pre-incubated with indicated PSC-CM or SFM, for 72 h. **E** Cytotoxicity assessment in PCCs pre-incubated with PSC-CM or SFM, and treated with GEM, NV-5440, or GEM + NV-5440. Data presented as mean ± SEM of four replicates. For **B-D**, **p < 0.01 comparing control and NV-5440 treated PCCs. For **E**, **p < 0.01 comparing basal with GEM or GEM + NV-5440. ^#^*p* < 0.05, ^##^*p* < 0.01 comparing GEM with GEM + NV-5440 in both SFM and PSC-CM. *PCC* pancreatic cancer cell, *PSC-CM* pancreatic stellate cell-conditioned medium, *SFM* serum-free DMEM

Next, it was investigated whether PSC-induced loss of GEM sensitivity in PCCs can be prevented by inhibition of glycolysis. As expected, exposure to NV-5440 inhibited glucose transport in a dose-dependent fashion in all PCC lines (Fig. 4A). Treatment with NV-5440 at a final concentration of 10 µM resulted in > 80% inhibition of glucose transport compared to DMSO controls in all PCC lines (*p* < 0.01; Fig. 4A). Among PCCs, NV-5440-induced inhibition of glucose transport was strongest in Mia PaCa-2 and weakest in SW-1990 (Fig. 4A). Furthermore, NV-5440-treated PCCs showed 2.0- to 2.6-fold (*p* < 0.05) lower extracellular lactate compared to untreated cells, with the highest and lowest effect seen in Panc-1 and HPAF-II, respectively (Fig. 4B). Next, PCCs pre-exposed to PSC-CMs were treated with NV-5440 and investigated for glucose transport, lactate release, and GEM sensitivity. Glucose transport, which varied among PCCs at baseline as well as following exposure to the various PSC-CMs, was significantly reduced by NV-5440 treatment across PCCs (Fig. 4C). Notably, inhibition of PSC-CM-induced glucose transport by NV-5440 was less prominent in PCCs exposed to HPaSteC as compared to PSC-1 and PSC-2 (Fig. 4C). Moreover, the PSC-CM-induced increase in the release of extracellular lactate by PCCs was significantly reduced by NV-5440, down to or below the levels observed for SFM (Fig. 4D). Comparison of the inhibition of lactate release by NV-5440 between various PSC-CMs revealed no significant differences. A significantly lower GEM-induced cytotoxicity was observed in PCCs exposed to PSC-CMs compared to SFM controls (Fig. 4E). Interestingly, this PSC-CM-induced loss of GEM sensitivity was not detected in PCCs treated with GEM + NV-5440. As such, in the presence of NV-5440, GEM-induced cytotoxicity was relatively similar among the three PSC-CMs and did not significantly differ from SFM (Fig. 4E).

### Silencing of LDHA and MCT4 inhibits glycolysis and promotes GEM sensitivity in PCCs

**Fig. 5:**
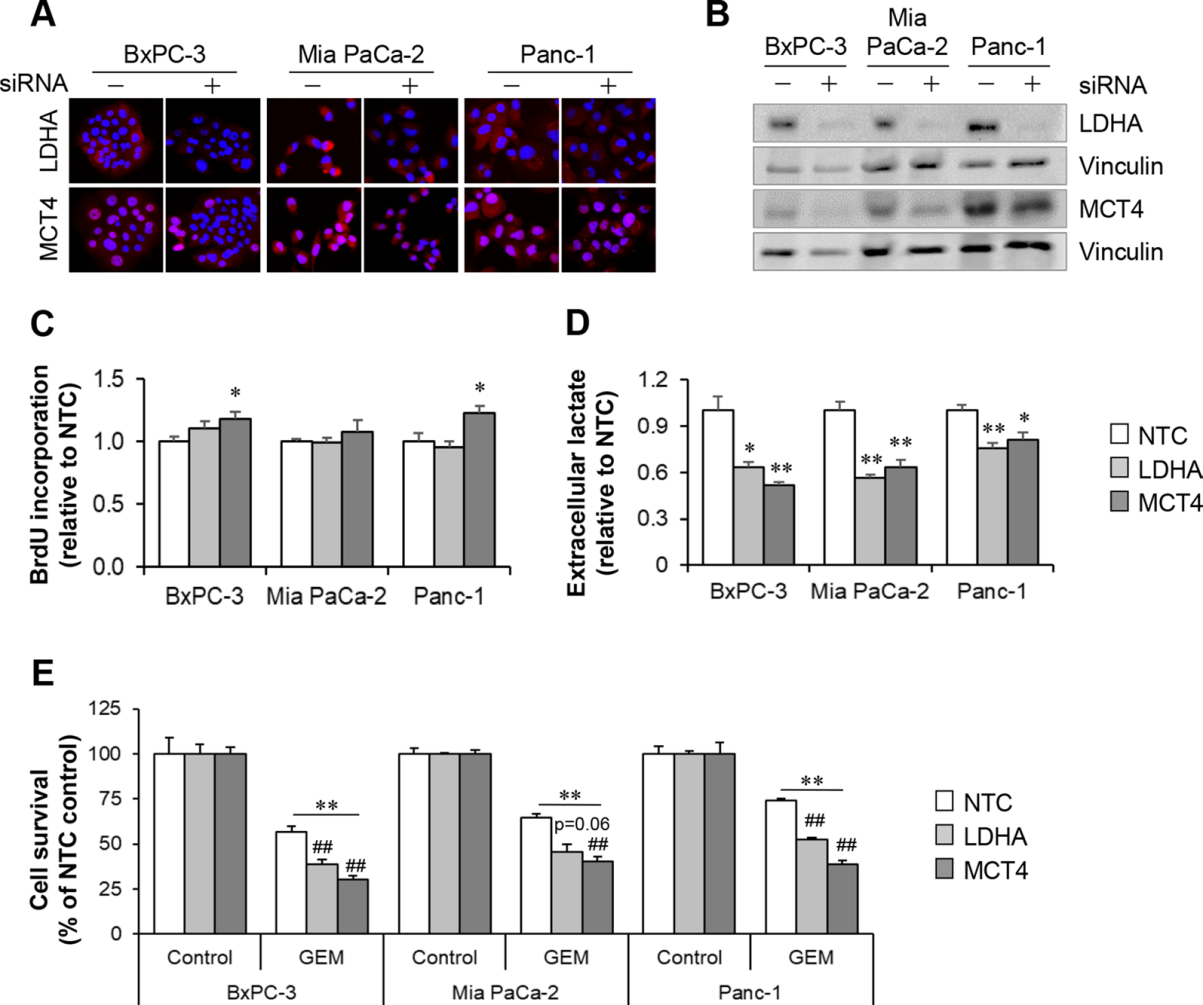
LDHA and MCT4 silencing promotes GEM sensitivity in PCCs. BxPC-3, Mia PaCa-2, and Panc-1 cells were transfected with NTC or siRNAs against LDHA and MCT4. Expression of LDHA and MCT4 was confirmed by **A** immunostaining and **B** western blot. Vinculin was used as loading control. Assessment of **C** proliferation, **D** extracellular lactate release, and **E** GEM sensitivity in PCCs transfected with NTC or siRNAs against LDHA and MCT4. Data presented as mean ± SEM of 4–6 replicates. For **C** and **D**, *p < 0.05, **p < 0.01 comparing NTC with LDHA or MCT4 transfected cells. For **E**, **p < 0.01 comparing control with GEM treated cells, and ^##^*p* < 0.01 comparing NTC with LDHA or MCT4 transfected cells. *NTC* negative transfection control, *LDHA* lactate dehydrogenase A, *MCT4* monocarboxylate transporter 4, *PCC* pancreatic cancer cell

Three PCC lines (BxPC-3, Mia PaCa-2, and Panc-1) transfected with NTC or siRNAs against LDHA and MCT4 were investigated for changes in proliferation, glycolysis, and GEM sensitivity. Successful gene silencing was confirmed by observed lower expression of LDHA and MCT4 in cells transfected with targeted siRNAs as compared to NTC (Fig. 5A, B). Transfection efficiency was confirmed using nuclear incorporation of siGLO (Additional file 2: Fig. S3). Reduction in MCT4 expression, following its silencing, varied among the three PCCs (Fig. 5B). LDHA silencing showed no impact on proliferation in any of the three PCCs, whereas a moderate increase in proliferation was observed upon MCT4 silencing in BxPC-3 and Panc-1 as compared to NTC (Fig. 5C). Silencing of LDHA and MCT4 resulted in significant reduction of extracellular lactate release in all three PCCs, compared to NTC (Fig. 5D). The lactate release in BxPC-3, Mia PaCa-2, and Panc-1 was reduced by 37%, 43%, and 25% upon LDHA silencing, and by 49%, 37%, and 19% upon MCT4 silencing, respectively (*p* < 0.05; Fig. 5D). Exposure of the transfected PCCs to GEM (10 µM) for 48 h resulted in significantly higher cell death in LDHA- and MCT4-silenced cells as compared to NTC (Fig. 5E). A trend towards higher GEM sensitivity was observed following silencing of MCT4 compared to LDHA, but differences were not statistically different.

### Analysis of PSC-CMs identified diverse protein expression profiles

**Fig. 6:**
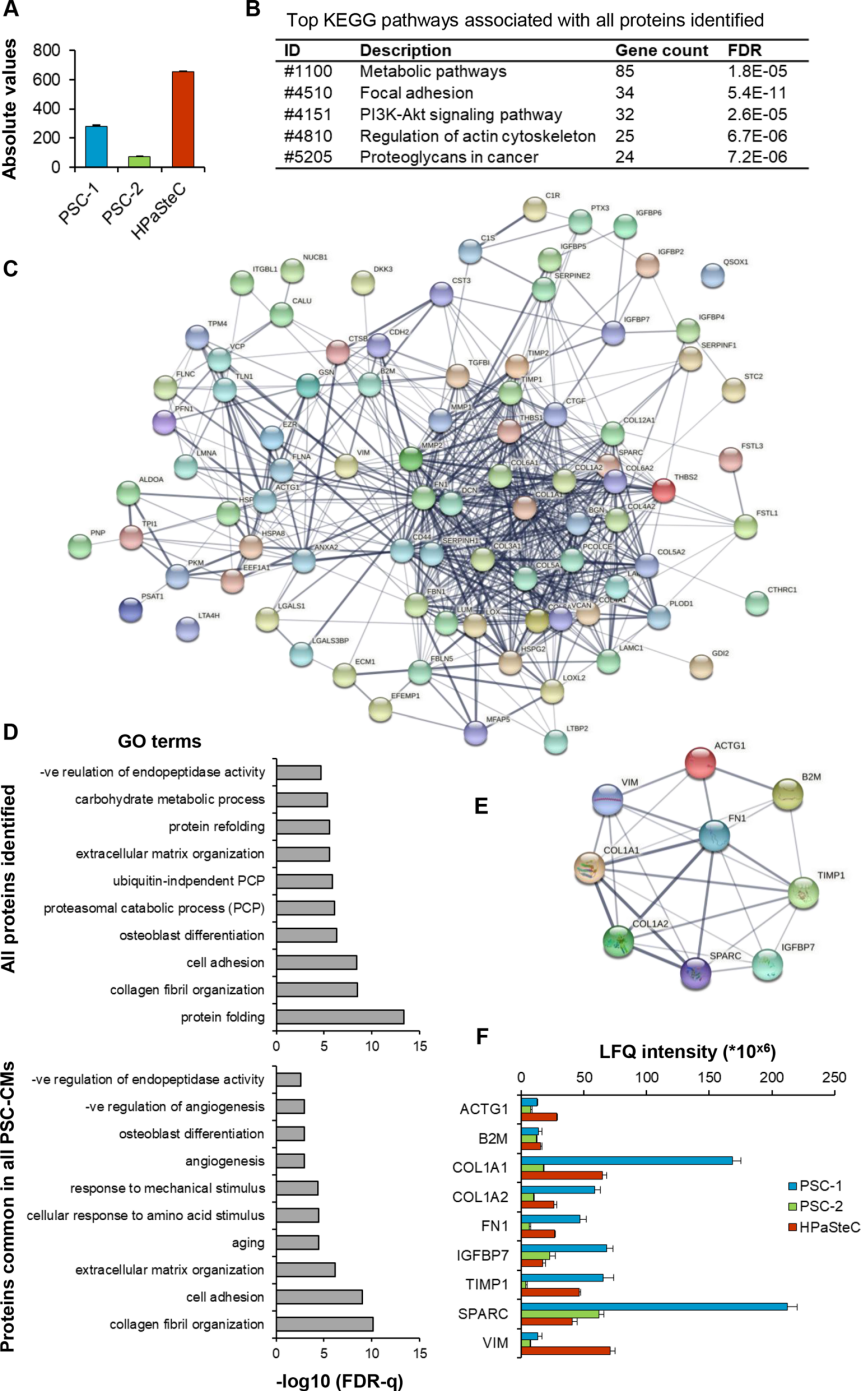
Analysis of pancreatic stellate cell-conditioned medium (PSC-CM). Three different PSC-CMs (PSC-1, PSC-2, and HPaSteC) were subjected to mass spectrometry, which identified 688 unique proteins. **A** Number of quantifiable proteins in each PSC-CM. **B** List of five major KEGG pathways associated with all proteins identified. **C** STRING network of 87 proteins expressed in all three PSC-CMs. **D** Functional annotation: gene ontology (GO) terms for frequently enriched biological processes, for all proteins identified (upper panel) and for proteins common to the three PSC-CMs (lower panel). **E** STRING network and **F** expression pattern of nine proteins in the top 25% of most expressed protein common to all three PSC-CMs. Data presented as mean ± SEM of three replicates in each PSC-CM

The MS analysis of PSC-CMs identified overall ~6600 peptides, mapping to a total of 688 unique proteins (Fig. 6A, Additional file 3). The protein composition and levels of expression differed between the three PSC-CMs. In addition, the number of quantifiable proteins differed: 674 in HPaSteC, 333 in PSC-1, and only 93 in PSC-2 (Fig. 6A). In total 194 proteins, accounting for 28.2% of all proteins identified, had a peptide count of > 10 each, while for the remaining > 70% proteins the peptide count was between 1 to 9 (Additional file 3). Notably, the highest peptide count of 110 was detected for fibronectin, followed by 101 and 100 peptides for plectin and fibrillin-1, respectively (Additional file 3). Pathway analysis of all proteins identified revealed their association with five major KEGG pathway terms: metabolic pathways, focal adhesion, PI3K-AKT signaling, regulation of actin cytoskeleton, and proteoglycans in cancer (Fig. 6B). Of all proteins identified, only 87 (i.e., 12.6%) were common to the three PSC-CMs. A STRING network of these proteins is shown in Fig. 6C, and the complete list is provided in Additional file 3. Functional annotation of frequently enriched GO-terms for biological processes by all proteins identified and by the proteins that are common to the three PSC-CMs, is presented in Fig. 6D. The most frequently enriched terms include collagen fibril organization, cell adhesion, protein binding, and extracellular matrix (ECM) organization (Fig. 6D). Next, all proteins were organized according to their expression level for each PSC-CM. Among the 25% most highly expressed proteins in each of the PSC-CMs, nine proteins were common to all three PSC-CMs: SPARC (secreted protein acidic and cysteine-rich), fibronectin, vimentin, collagen 1A1, collagen 1A2, B2M (beta-2-microglobulin), ACTG1 (actin, cytoplasmic 2), TIMP1 (metalloproteinase inhibitor 1), and IGFBP7 (insulin-like growth factor-binding protein 7). The majority of these proteins are ECM-associated and closely linked, as shown in the STRING network (Fig. 6E). The expression patterns of these proteins indicate considerable heterogeneity between the three PSC-CMs (Fig. 6F). The protein with the highest expression level in the CM from both PSC-1 and PSC-2 was SPARC, whereas this was vimentin for HPaSteC (Fig. 6F, Additional file 3).

### PSC-CM-induced enhanced glycolysis and reduced sensitivity to GEM are mediated by ERK phosphorylation

**Fig. 7:**
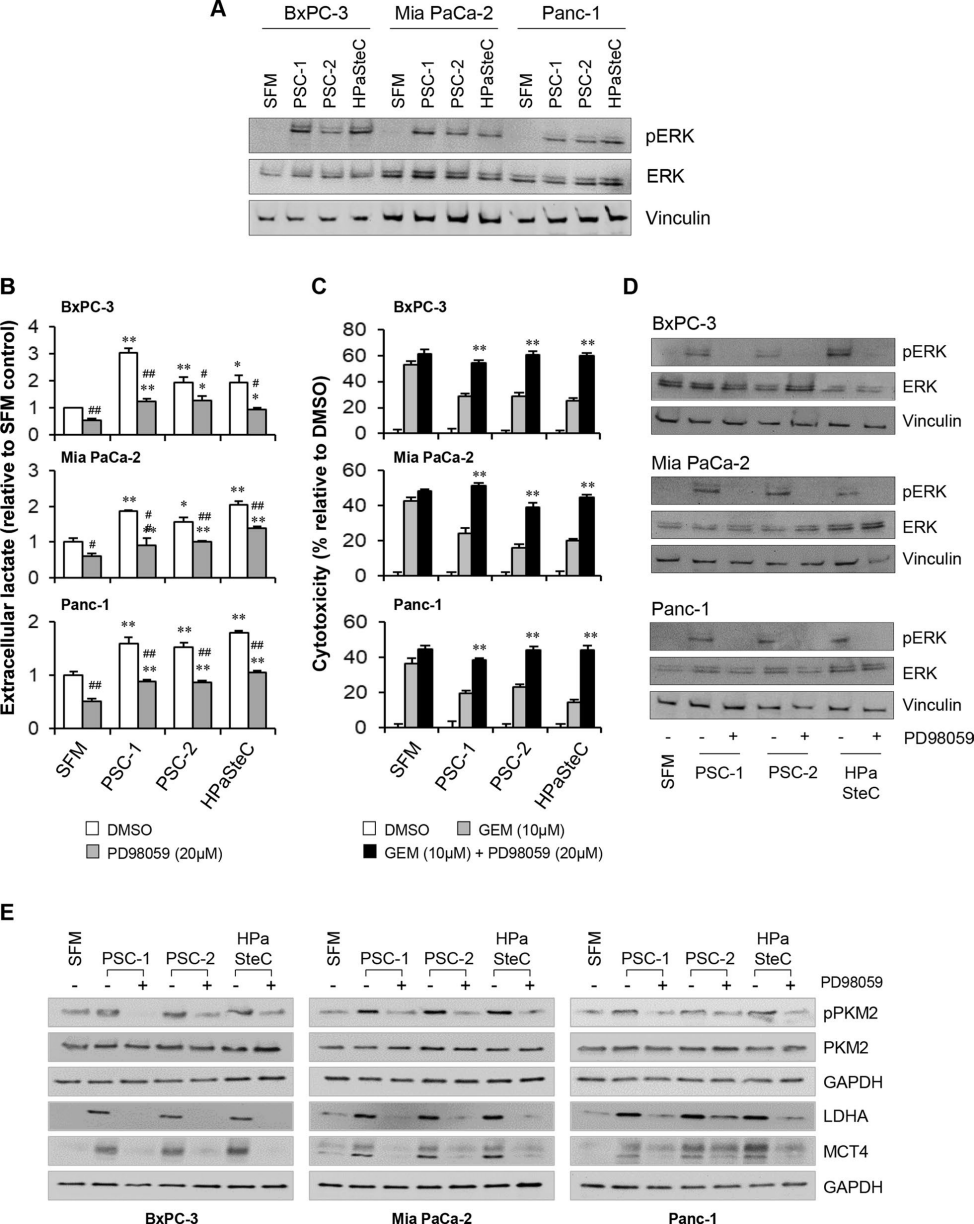
Effect of ERK inhibitor PD98059 on glycolysis and GEM sensitivity in PCCs. PCCs pre-incubated for 72 h with three different PSC-CMs (PSC-1, PSC-2, and HPaSteC) were assessed for **A** expression of pERK/ERK, **B** extracellular lactate release, following treatment with PD98059, **C** cytotoxicity following treatment with GEM or GEM + PD98059, and **D**, **E** expression of indicated markers following treatment with PD98059. Vinculin (**A**, **D**) and GAPDH (**B**) were used as a loading control. For **B**, *p < 0.05, **p < 0.01 comparing SFM vs PSC-CMs and #*p* < 0.05, ^##^*p* < 0.01 comparing DMSO vs PD98059 treated. For **C** **p < 0.01 comparing GEM vs GEM + PD98059. Data presented as mean ± SEM of three replicates in each PSC-CM. *LDHA* lactate dehydrogenase A, *MCT4* monocarboxylate transporter 4, *PKM2* pyruvate kinase M2, *PCCs* pancreatic cancer cells, *PSCs* pancreatic stellate cells

Increased expression of phosphorylated ERK was observed in PCCs (BxPC-3, Mia PaCa-2, and Panc-1) following their exposure to the three different PSC-CMs for 72 h (Fig. 7A). Next, when PCCs pre-incubated with PSC-CMs were treated with the MEK/ERK inhibitor PD98059 (20 µM), there was a significant reduction in PSC-induced lactate release by PCCs as compared to untreated controls (Fig. 7B). Furthermore, a significant increase in the cytotoxic response of GEM was observed when GEM was added in combination with PD98059 to PCCs pre-incubated with PSC-CMs (Fig. 7C). The PD98059-induced inhibition of phosphorylated ERK expression was confirmed by western blot analysis (Fig. 7D). Lastly, increased levels of phosphorylated PKM2, LDHA and MCT4 were observed in PCCs exposed to PSC-CMs for 72 h, which were reduced following treatment with PD98059 (Fig. 7E).

## Discussion

For more than two decades, GEM has been essential in the treatment of PDAC, however, its clinical benefits are typically limited by profound chemoresistance. Resistance to GEM in PDAC is multifactorial, and the exact underlying mechanisms are hitherto unknown. PSCs and metabolic alterations are important for tumor growth and both are increasingly suggested to affect GEM sensitivity in PDAC [8, 13, 20, 30, 31]. A major metabolic alteration in PDAC is the shift from oxidative phosphorylation to glycolysis, which recently was shown to promote GEM resistance [20, 31]. The Warburg effect, i.e., a dramatically increased glucose transport and glycolytic flux even in the presence of oxygen and normal mitochondrial function, is a prominent feature of PDAC [32, 33]. However, it remains unknown whether the effects of PSCs on GEM sensitivity in PCCs are associated with the regulation of glycolysis. Investigation of the latter was the aim of this study.

First, the effects of PSCs on various steps in the glycolytic pathway in PCCs were investigated by the exposure of the latter to PSC-CMs. As neoadjuvant chemotherapy is increasingly used for the treatment of PDAC but the impact on the PSCs and their interactions with the PCCs is currently unknown, conditioned medium from PSCs derived from both treatment-naïve and NAT-treated PDAC were included in the experiments. Glucose transport in PCCs did not seem to be affected by PSC-CM, although the expression of GLUT1 was increased in three of the PCC lines. Interestingly, increased GLUT1 expression is reported to be associated with poor prognosis in PDAC [34, 35]. Of the enzymes that catalyze the fermentation of glucose to lactate, three key regulators were investigated: HK2 and PKM2, which drive the first and final step of glycolysis, respectively, and LDHA, which controls the conversion of pyruvate to lactate. While exposure to PSC-CMs had a markedly variable effect on HK2, it resulted in a significantly increased expression of PKM2 and LDHA in four and all of the PCCs, respectively. Moreover, expression of MCT4, which exports lactate to the extracellular space, was also significantly increased in all PCCs following exposure to any of the PSC-CMs. Of note, the expression of HK2 and PKM2 has been previously reported to promote PDAC growth [23, 36, 37]. Similarly, LDHA and MCT4 are known to be associated with enhanced glycolytic metabolism and poor prognosis of PDAC [25, 38, 39]. The present study reveals that exposure to PSC-CM induces a concerted increase in several of the glycolysis regulators. In particular, LDHA and MCT4 were increased in all six PCC lines that were tested, and PKM2 was increased in four of the PCCs. Taken together, these findings indicate that exposure to PSC-CM induces a glycolytic phenotype in PCCs, which is known to be pro-tumorigenic and associated with poor prognosis in PDAC [25, 39, 40].

In the next set of experiments, it was shown that exposure to PSC-CM reduces the sensitivity for GEM in PCCs, which is in accordance with previous data published by ourselves and others [8, 12, 41]. To investigate whether GEM resistance in PCCs is dependent on glycolytic activity, the glycolytic pathway in PCCs was experimentally inhibited, either pharmacologically using NV-5440 or by transient gene silencing of LDHA and MCT4. NV-5440 is a small molecular inhibitor of glucose transport and glycolysis, which acts by selective inhibition of GLUT1 and the mTORC1 pathway [42]. Treatment with NV-5440 nearly blocked glucose transport and approximately halved lactate release, both at baseline and following induction by PSC-CM exposure. Importantly, NV-5440 restored the PSC-CM-induced reduction of GEM cytotoxicity in PCCs. Similarly, silencing LDHA or MCT4, which was confirmed to reduce extracellular lactate levels, increased GEM-induced cytotoxicity in PCCs. These results indicate that activity throughout the entire glycolysis pathway, from glucose uptake to lactate production and secretion, is associated with increased GEM resistance in PCCs. This is in line with the growing evidence that supports glycolysis-mediated GEM resistance in PDAC [19–22].

**Fig. 8:**
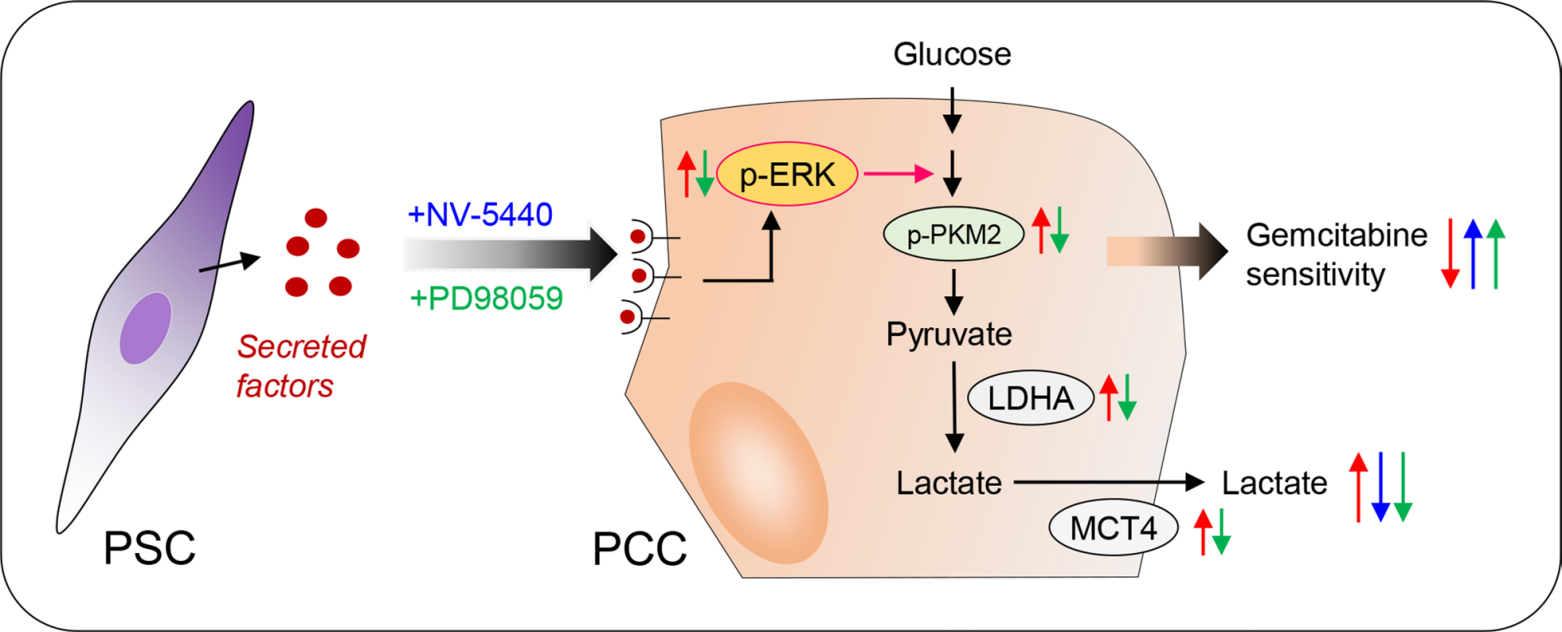
Impact of PSCs on glycolysis and GEM sensitivity in PCCs. PSC-secreted factors promote glycolysis and induce loss of GEM sensitivity through increased ERK phosphorylation. Glycolysis in PCCs is mediated by increased expression of LDHA and MCT4. Exposure to inhibitors of glycolysis (NV-5440) or MEK/ERK (PD98059) showed protection of PCCs from both PSC-induced loss of GEM sensitivity and enhanced glycolysis as indicated by the increased secretion of lactate. *GEM* gemcitabine, *LDHA* lactate dehydrogenase A, *MCT4* monocarboxylate transporter 4, *PCCs* pancreatic cancer cells, *PKM2* pyruvate kinase M2, *PSCs* pancreatic stellate cells

In a final series of experiments aiming at identifying PSC-secretory proteins that are potentially responsible for the observed changes in glycolytic activity and GEM sensitivity, PSC-CMs were analyzed by MS. Although the number of quantifiable proteins varied significantly between the three PSC-CMs, the majority of highly expressed PSC-secreted proteins were ECM related, including fibronectin, collagen 1A1, collagen 1A2, and SPARC, all of which have been shown to modulate GEM sensitivity in PDAC [8, 43–45]. Increased ERK activity has been reported to contribute to GEM resistance in PCCs [8, 46, 47]. Activation of ERK is frequently observed in multiple cancers and has been shown to promote the Warburg effect, particularly via ERK-dependent phosphorylation and nuclear translocation of PKM2 [48, 49]. Moreover, PKM2 expression and activity is suggested to promote chemoresistance [50–53]. In the present study, we observed increased ERK and PKM2 phosphorylation combined with increased LDHA and MCT4 expression in PCCs following their exposure to PSC-CMs. Furthermore, following the incubation of PCCs with PSC-CMs, treatment of these PCCs with PD98059, an inhibitor of MEK/ERK, resulted in a reduction of PSC-CM-induced glycolysis and GEM resistance. Taken together, these findings suggest that PSC-secreted factors induce increased ERK phosphorylation, which enhances glycolytic activity through increased PKM2 phosphorylation and expression of LDHA and MCT4, ultimately promoting GEM resistance in PCCs (Fig. 8).

The findings of this study also reveal marked heterogeneity among the six PCC lines that were tested, both in terms of glycolytic activity, GEM sensitivity, and the effects of the different PSC-CMs. Variation of the latter is likely related to the marked difference in secreted protein composition that was observed, both qualitatively and quantitatively. Differences in the origin of the PSCs—human PDAC (PSC-1, PSC-2) and normal human fetal pancreas (HPaSteC)—may be a likely, although not exclusive, explanation for the observed functional heterogeneity among the PSCs, as previously described by our group [29]. Heterogeneity among the various PCCs, both at baseline and following exposure to PSC-CMs, may be related to the genotypic and phenotypic differences that are well-described for these cell lines [54]. While the impact of PSC-CMs on the glycolytic activity and GEM resistance in PCCs is consistent and significant, the emerging picture is complex due to heterogeneity in both the cancer cell and pancreatic stellate cell populations as well as in their mutual interactions. The findings of this study suggest that the glycolytic pathway may be a target to improve GEM sensitivity, although the observed complex heterogeneity represents a challenge for translation into precision medicine.

The study has several limitations. First, given the marked heterogeneity both among the six PCCs and the three PSC-CMs that were investigated, the observations made in this study may require confirmation by testing a large panel of cell cultures, including primary PDAC cell cultures. Second, transient gene silencing results, by nature, in the suppression of protein expression for only a limited duration, but was used in this study as PCC lines with stable knockdown for the genes of interest were unavailable. Third, from the multitude of secretory proteins that are contained in PSC-CMs, the study did not identify a single factor that may be responsible for the observed effects of PSC-CM exposure on glycolysis and GEM cytotoxicity in PCCs. Lastly, the experimental setting is purely limited to in vitro analysis using immortalized PCCs, which may not fully reflect the conditions that govern the processes in human PDAC in vivo.

## Conclusions

Exposure to PSC-CM promotes proliferation, glycolysis, and GEM resistance in PCCs, while glucose transport was unchanged. The PSC-induced GEM resistance in PCCs was glycolysis-dependent and mediated by the upregulation of ERK phosphorylation. As such, inhibition of glycolysis by NV-5440, showed protection from the impact of PSC-CMs, while silencing of key glycolysis regulators—LDHA and MCT4—as well as the inhibition of ERK by PD98059, improved GEM sensitivity. Of note, this is the first study to demonstrate the possible therapeutic potential of NV-5440 through its glycolysis inhibitory effect in PDAC. Lastly, this study provides new evidence supporting the growing concept of glycolysis-mediated GEM resistance in PDAC and the role of PSCs in this process. Further investigations are needed to better understand the molecular relations between glycolytic alterations and GEM sensitivity in PDAC.

## Supplementary Information

**Additional file 1: Table S1.** Cell lines information. **Table S2.** Clinical information. **Table S3.** Primer sequences of stealth RNAi siRNAs. **Table S4.** Antibody information.

**Additional file 2: Fig. S1.** Representative pictures of pancreatic stellate cells (PSCs) immunostained with smooth muscle actin (α -SMA; Green) and Vimentin (Red). Nuclei stained with DAPI (blue). **Fig. S2.** Representative pictures of pancreatic cancer cells stained with crystal violet, following their incubation with SFM or PSC for 72h. PSC pancreatic stellate cells; PSC-CM, PSC-conditioned medium; SFM, serum-free DMEM. **Fig. S3.** Immunofluorescence images of PCCs transfected with siGLO using Lipofectamine RNAiMAX reagent. PCC, pancreatic cancer cells.

**Additional file 3**. PSC-secretome data.

**Additional file 4**. Western blot images.

## Data Availability

The datasets used and/or analyzed during the current study are available from the corresponding author on reasonable requests.

## Abbreviations

ECM: Extracellular matrix
GEM: Gemcitabine
GLUT1: Glucose transporter 1
HK: Hexokinase
LDHA: Lactate dehydrogenase A
MCT: Monocarboxylate transporter
NTC: Negative transfection control
PCC: Pancreatic cancer cell
PDAC: Pancreatic ductal adenocarcinoma
PKM2: Pyruvate kinase M2
PSC: Pancreatic stellate cell
PSC-CM: Pancreatic stellate cell-conditioned medium
SFM: Serum-free DMEM
TME: Tumor microenvironment
