# Supplementary material for: Pancreatic stellate cell-induced gemcitabine resistance in pancreatic cancer is associated with LDHA- and MCT4-mediated enhanced glycolysis
Source: Cancer Cell Int. 2023 Jan 19;23:9. doi: 10.1186/s12935-023-02852-7 (PMC9850604; doi:10.1186/s12935-023-02852-7)
Supplement: Supplementary file 1 — Additional file 1: Table S1. Cell lines information. Table S2. Clinical information. Table S3. Primer sequences of stealth RNAi siRNAs. Table S4. Antibody information. [file 12935_2023_2852_MOESM1_ESM.pdf]

Additional File 1: Table S1, S2, S3, and S4

Table S1: Cell lines information

Cell line	Doubling time (h)	Common mutations
BxPC-3	48-60	*KRAS*: WT, *TP53*: 220Cys, *p16*: WT, *SMAD4*: HD
Capan-2	96	*KRAS*: 12Val, *TP53*: WT, *p16*: WT, *SMAD4*: WT
HPAF-II	42	*KRAS*: 12Asp, *TP53*: 151Ser, *p16*: WT, *SMAD4*: WT
Mia PaCa-2	40	*KRAS*: 12Cys, *TP53*: 248Trp, *p16*: Δ20–25, *SMAD4*: WT
Panc-1	53	*KRAS*: 12Asp, *TP53*: 272His, *p16*: HD, *SMAD4*: WT
SW-1990	64	*KRAS*: 12Asp, *TP53*: WT, *p16*: WT, *SMAD4*: WT

WT, Wild type; HD, homozygous deletion; Δ, deletion.

Table S2: Clinical information

Cell line	Source PDAC	Tumor size (mm)	Treatment	Survival (months)
PSC-1	Treatment naïve	30 mm	-	20.2
PSC-2	Neoadjuavntly-treated	47 mm	Folfirinox x4	21.3

PDAC, pancreatic ductal adenocarcinoma; PSC, pancreatic stellate cell.

Table S3: Primer sequences of stealth RNAi siRNAs

Target	siRNA sequence	Cat #, Supplier
LDHA	5’-UGUAGCAGAUUUGGCAGAGAGUAUA-3’	HSS106002
	5’-UAUACUCUCUGCCAAAUCUGCUACA-3’	Thermo Fisher Scientific
MCT4	5’-CCUCGCUCAUCAUGCUGAACCGCUA-3’	HSS145028
	5’-UAGCGGUUCAGCAUGAUGAGCGAGG-3’	Thermo Fisher Scientific

Table S4: Antibody information

Target	Supplier	Cat #	RRID
α-SMA	Nordic Biosite AB	BSH-7459	not available
Vimentin	Cell Signaling Tech.	5741	BDSC_5741
Glycolysis	Cell Signaling Tech.	8337	AB_10897509
GLUT1	Sigma-Aldrich	SAB4200519	not available
MCT1	Abcam	ab85021	AB_10674945
MCT4	Abcam	ab234728	not available
pERK	Cell Signaling Tech.	4370	AB_2315112
ERK	Cell Signaling Tech.	4695	AB_390779
Vinculin	Cell Signaling Tech.	13901	AB_2728768
Phospho-PKM2 (Ser37)	ThermoFisherScientific	PA5-37684	AB_2554292
Immpress HRP Anti-Rabbit	Vector Labs	MP-7401	AB_2336529
Immpress HRP Anti-Mouse	Vector Labs	MP-7402	AB_2336528
HRP-Goat Anti-Rabbit	Bio-Rad Labs	1706515	AB_11125142

α-SMA, alpha-smooth muscle actin; GLUT1, glucose transporter 1; HRP, Horseradish peroxidase; MCT, monocarboxylate transporter; PKM2, pyruvate kinase M2.
