# Supplementary material for: Pancreatic stellate cell-induced gemcitabine resistance in pancreatic cancer is associated with LDHA- and MCT4-mediated enhanced glycolysis
Source: Cancer Cell Int. 2023 Jan 19;23:9. doi: 10.1186/s12935-023-02852-7 (PMC9850604; doi:10.1186/s12935-023-02852-7)
Supplement: Supplementary file 2 — Additional file 2: Fig. S1. Representative pictures of pancreatic stellate cells (PSCs) immunostained with smooth muscle actin (α -SMA; Green) and Vimentin (Red). Nuclei stained with DAPI (blue). Fig. S2. Representative pictures of pancreatic cancer cells stained with crystal violet, following their incubation with SFM or PSC for 72h. PSC pancreatic stellate cells; PSC-CM, PSC-conditioned medium; SFM, serum-free DMEM. Fig. S3. Immunofluorescence images of PCCs transfected with siGLO using Lipofectamine RNAiMAX reagent. PCC, pancreatic cancer cells. [file 12935_2023_2852_MOESM2_ESM.pdf]

Additional File 2: Fig. S1, S2, and S3

Fig. S1

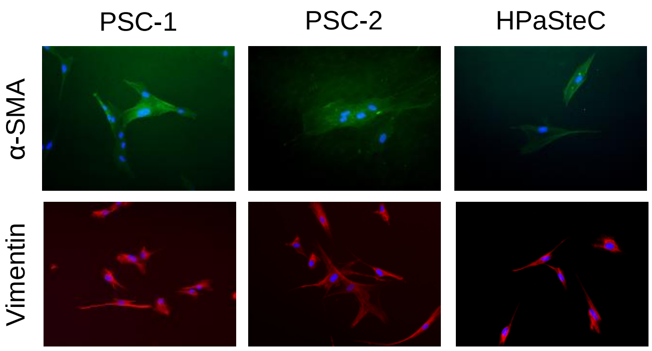

Fig. S1. Representative pictures of pancreatic stellate cells (PSCs) immunostained with smooth muscle actin (α-SMA; Green) and Vimentin (Red). Nuclei stained with DAPI (blue).

Fig. S2

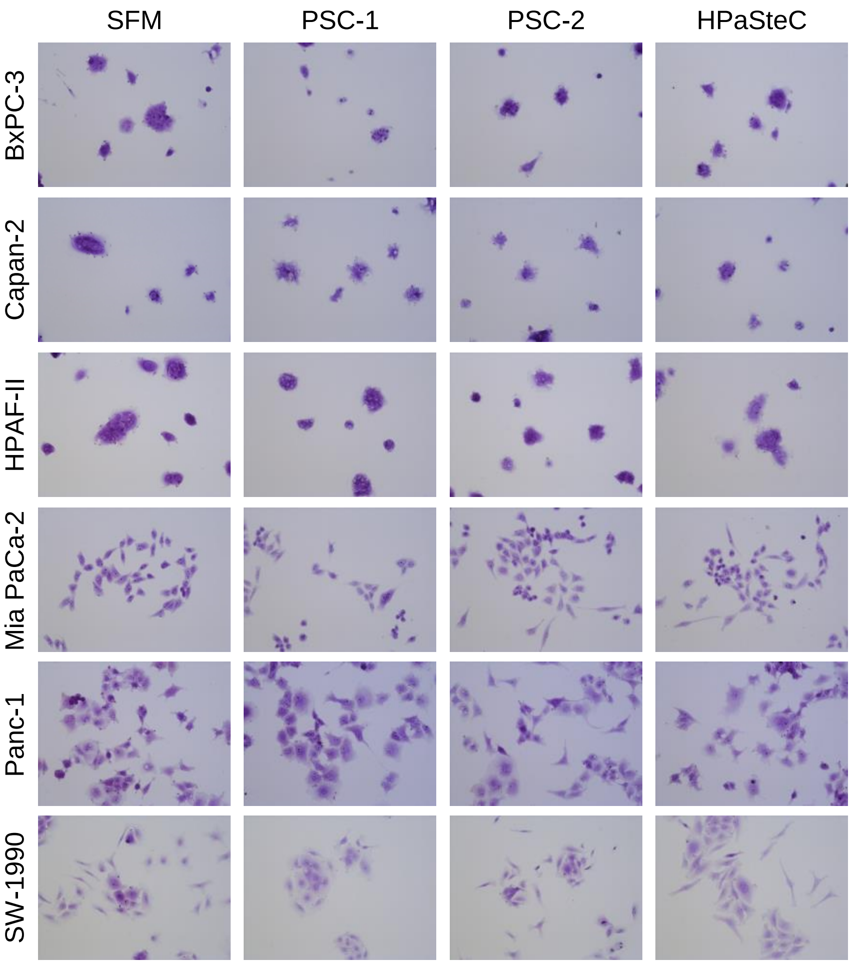

Fig. S2. Representative pictures of pancreatic cancer cells stained with crystal violet, following their incubation with SFM or PSC-CM for 72h. PSC, pancreatic stellate cells; PSC-CM, PSC-conditioned medium; SFM, serum-free DMEM.

Fig. S3

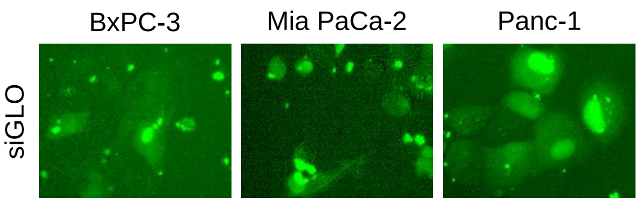

Fig. S3. Immunofluorescence images of PCCs transfected with siGLO using Lipofectamine RNAiMAX reagent. PCC, pancreatic cancer cells
