# Supplementary figures and images for: Pancreatic stellate cell-induced gemcitabine resistance in pancreatic cancer is associated with LDHA- and MCT4-mediated enhanced glycolysis
Source: Cancer Cell Int. 2023 Jan 19;23:9. doi: 10.1186/s12935-023-02852-7 (PMC9850604; doi:10.1186/s12935-023-02852-7)

# Additional file 4

Blots Fig. 3B

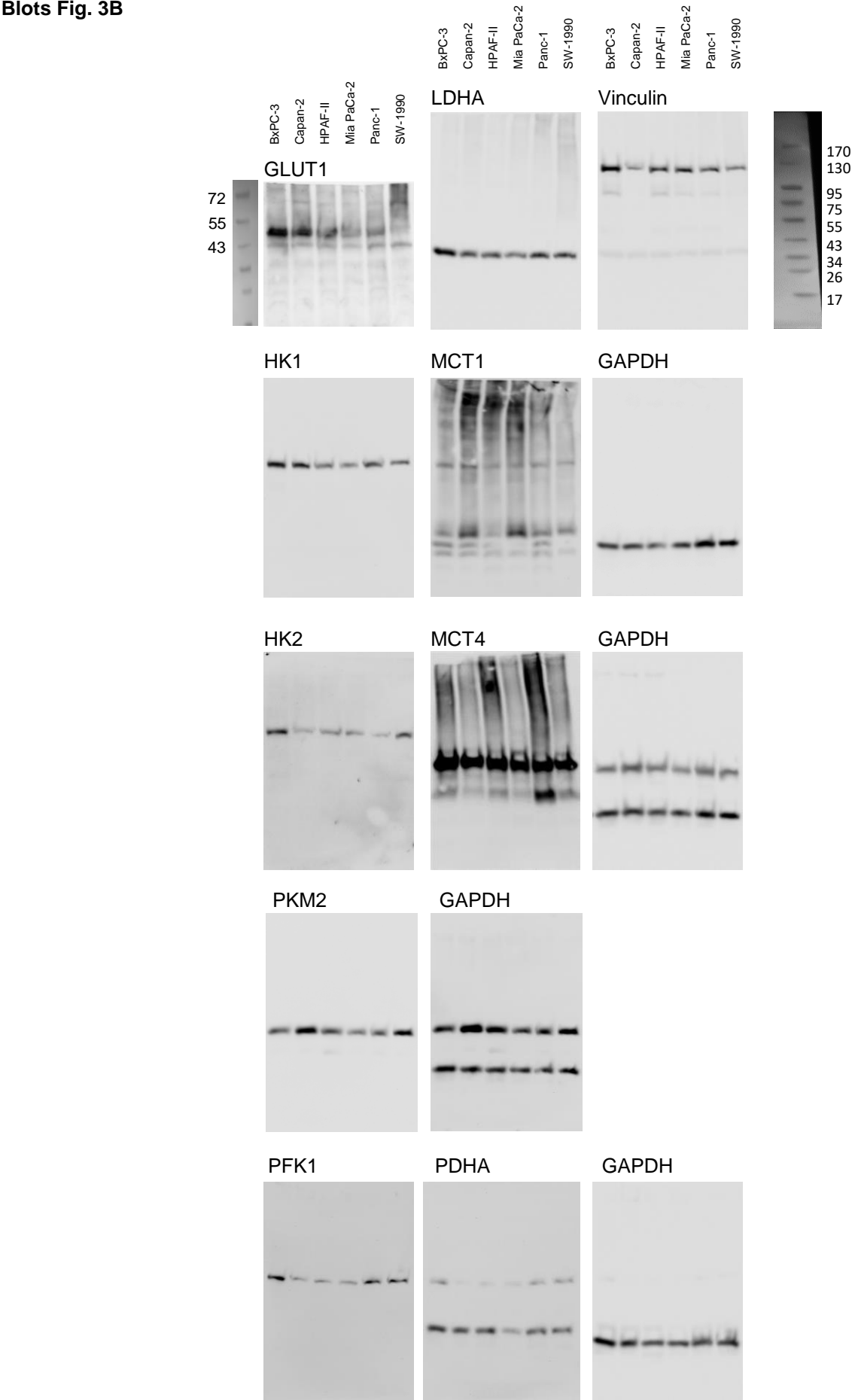

Blots Fig. 3C

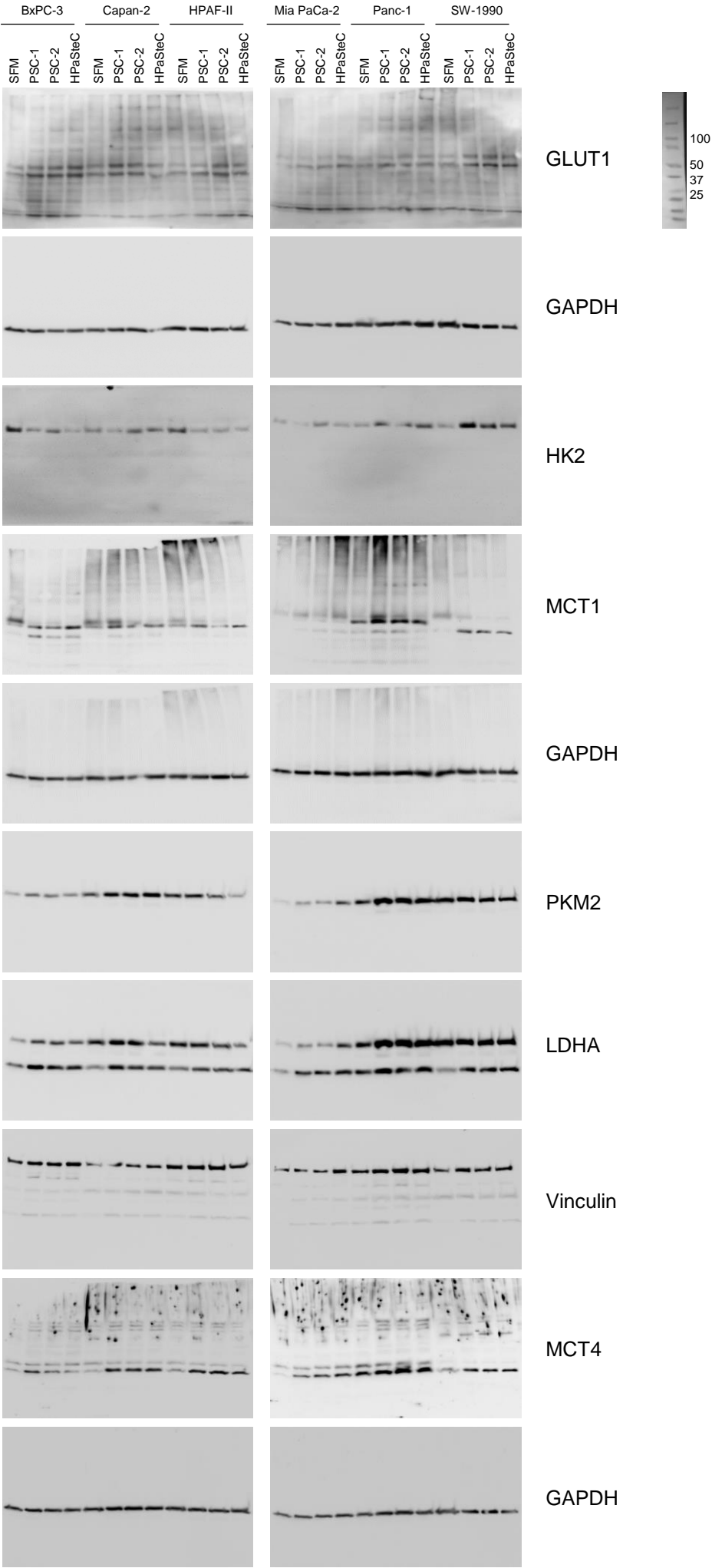

Blots Fig. 5B

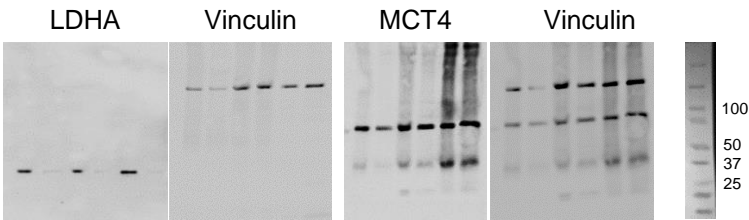

Blots Fig. 7A

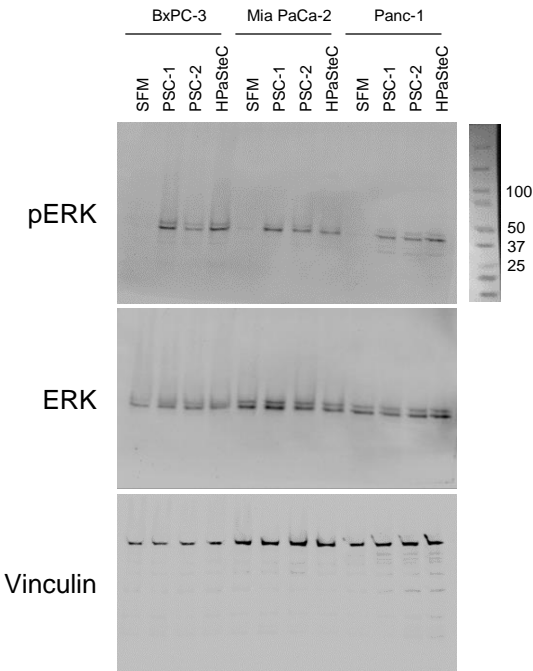

Blots Fig. 7D

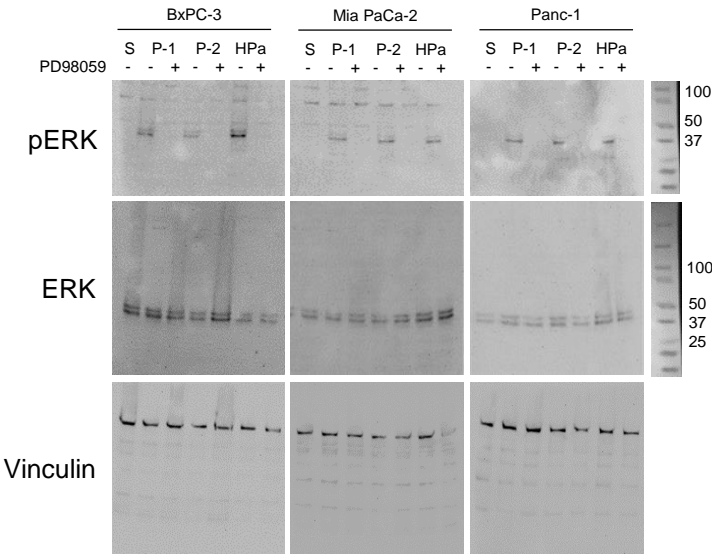

Blots Fig. 7E

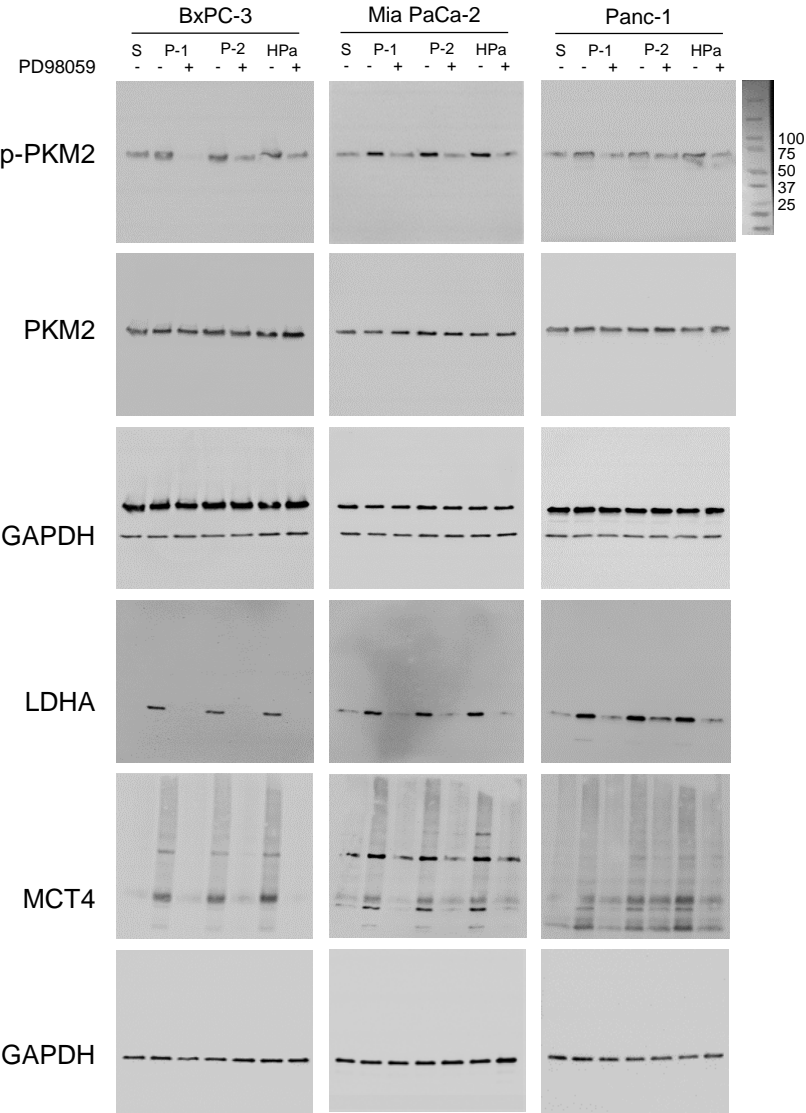

Supplement: Supplementary file 4 — Additional file 4. Western blot images. [file 12935_2023_2852_MOESM4_ESM.pdf]
